# 6-Bromo-1,3-benzothia­zol-2-amine

**DOI:** 10.1107/S1600536812040123

**Published:** 2012-09-26

**Authors:** Xing-Jun Gao, Shou-Wen Jin, Yan-Fei Huang, Yong Zhou, Ying-Ping Zhou

**Affiliations:** a Faculty of Science, ZheJiang A & F University, Lin’An 311300, People’s Republic of China; b Tianmu College of ZheJiang A & F University, Lin’An 311300, People’s Republic of China

## Abstract

The r.m.s. deviation from the mean plane for the non-H atoms in the title compound, C_7_H_5_BrN_2_S, is 0.011 Å. In the crystal, the mol­ecules are linked by N—H⋯N and N—H⋯Br hydrogen bonds to generate (010) sheets. Weak aromatic π–π stacking [centroid-to-centroid separation = 3.884 (10) Å] and possible C—H⋯Br inter­actions are also observed. The crystal studied was found to be an inversion twin.

## Related literature

For a related structure and background to benzothia­zole derivatives, see: Jin *et al.* (2012 ▶).
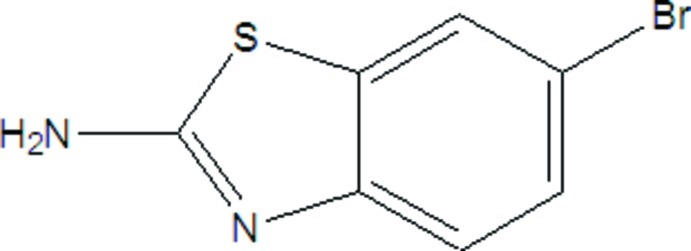


## Experimental

### 

#### Crystal data

C_7_H_5_BrN_2_S
*M* *_r_* = 229.10
Orthorhombic, 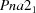

*a* = 8.6268 (7) Å
*b* = 22.487 (2) Å
*c* = 4.0585 (3) Å
*V* = 787.30 (11) Å^3^
*Z* = 4
Mo *K*α radiation
μ = 5.41 mm^−1^
*T* = 298 K
0.31 × 0.25 × 0.16 mm

#### Data collection

Bruker SMART CCD diffractometer
Absorption correction: multi-scan (*SADABS*; Bruker, 2002 ▶) *T*_min_ = 0.215, *T*_max_ = 0.421
3645 measured reflections
1340 independent reflections
929 reflections with *I* > 2σ(*I*)
*R*_int_ = 0.087

#### Refinement

*R*[*F* ^2^ > 2σ(*F* ^2^)] = 0.095
*wR*(*F* ^2^) = 0.246
*S* = 1.07
1340 reflections
100 parameters
1 restraint
H-atom parameters constrained
Δρ_max_ = 1.13 e Å^−3^
Δρ_min_ = −0.74 e Å^−3^
Absolute structure: Flack (1983 ▶), 539 Friedel pairs
Flack parameter: 0.42 (9)

### 

Data collection: *SMART* (Bruker, 2002 ▶); cell refinement: *SAINT* (Bruker, 2002 ▶); data reduction: *SAINT*; program(s) used to solve structure: *SHELXS97* (Sheldrick, 2008 ▶); program(s) used to refine structure: *SHELXL97* (Sheldrick, 2008 ▶); molecular graphics: *SHELXTL* (Sheldrick, 2008 ▶); software used to prepare material for publication: *SHELXTL*.

## Supplementary Material

Crystal structure: contains datablock(s) global, I. DOI: 10.1107/S1600536812040123/hb6956sup1.cif

Structure factors: contains datablock(s) I. DOI: 10.1107/S1600536812040123/hb6956Isup2.hkl

Supplementary material file. DOI: 10.1107/S1600536812040123/hb6956Isup3.cml

Additional supplementary materials:  crystallographic information; 3D view; checkCIF report

## Figures and Tables

**Table 1:** Hydrogen-bond geometry (Å, °)

*D*—H⋯*A*	*D*—H	H⋯*A*	*D*⋯*A*	*D*—H⋯*A*
N2—H2*B*⋯Br1^i^	0.86	3.04	3.864 (17)	160
N2—H2*A*⋯N1^ii^	0.86	2.26	2.94 (2)	136
C4—H4⋯Br1^iii^	0.93	2.87	3.402 (17)	118

Symmetry codes: (i) 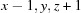; (ii) 
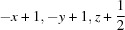
; (iii) 
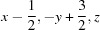
.

**Fig. 1.**
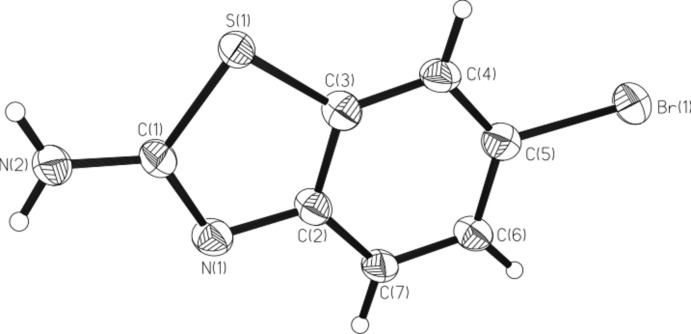
The structure of the title compound, showing displacement ellipsoids drawn at the 30% probability level.

**Fig. 2.**
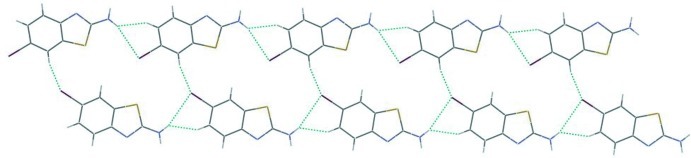
One-dimensional double chain structure.

## supplementary crystallographic information

### Comment

As an extension of our study of 2-aminoheterocyclic compounds (Jin *et al.*, 2012), herein we report the crystal structure of 6-bromobenzo[*d*]thiazol-2-amine.

The single crystals of the title compound (Fig.1) with the formula C_7_H_5_BrN_2_S was obtained by slow evaporating its methanol solution.

The 6-bromobenzo[*d*]thiazol-2-amine molecules (Fig. 1) are linked together in head to tail fashion *via* the N—H···Br association to form one-dimensional chain running along the direction that made a dihedral angle of *ca* 30° with the *a* axis direction. Two neighboring chains were held together by the CH—Br interaction with C—Br distance of 3.402 Å generating one-dimensional double chain (Fig.2). The double chains were stacked along the direction that is perpendicular with its extending direction by the CH—Br interaction with C—Br distance of 3.402 Å to form two-dimensional sheet extending parallel to the *ac* plane. The sheets were further stacked along the *b* axis direction by the intersheet N—H···N hydrogen bonds to form three-dimensional ABAB layer network structure.

### Experimental

The 6-bromobenzo[*d*]thiazol-2-amine (22.9 mg, 0.1 mmol) was dissolved in a methanol solution (8 ml). The solution was filtered into a test tube. The solution was left standing at room temperature for a month, light-yellow blocks were isolated after slow evaporation of the methanol solution to *ca* 3 ml in air.

### Refinement

H atoms bonded N atoms were located in a6-Bromo-1,3-benzothiazol-2-amine difference Fourier map and refined isotropically. Other H atoms were positioned geometrically with C—H = 0.93 Å, and constrained to ride on their parent atoms with *U*_iso_(H) = 1.2Ueq(C).

### Crystal data

**Table:**

C_7_H_5_BrN_2_S	*D*_x_ = 1.933 Mg m^−^^3^
*M**_r_* = 229.10	Mo *K*α radiation, λ = 0.71073 Å
Orthorhombic, *P**n**a*2_1_	Cell parameters from 868 reflections
*a* = 8.6268 (7) Å	θ = 3.6–21.4°
*b* = 22.487 (2) Å	µ = 5.41 mm^−^^1^
*c* = 4.0585 (3) Å	*T* = 298 K
*V* = 787.30 (11) Å^3^	Block, colourless
*Z* = 4	0.31 × 0.25 × 0.16 mm
*F*(000) = 448	

### Data collection

**Table:**

Bruker SMART CCD diffractometer	1340 independent reflections
Radiation source: fine-focus sealed tube	929 reflections with *I* > 2σ(*I*)
Graphite monochromator	*R*_int_ = 0.087
ω scans	θ_max_ = 25.0°, θ_min_ = 2.5°
Absorption correction: multi-scan (*SADABS*; Bruker, 2002)	*h* = −6→10
*T*_min_ = 0.215, *T*_max_ = 0.421	*k* = −26→26
3645 measured reflections	*l* = −4→4

### Refinement

**Table:**

Refinement on *F*^2^	Secondary atom site location: difference Fourier map
Least-squares matrix: full	Hydrogen site location: inferred from neighbouring sites
*R*[*F*^2^ > 2σ(*F*^2^)] = 0.095	H-atom parameters constrained
*wR*(*F*^2^) = 0.246	*w* = 1/[σ^2^(*F*_o_^2^) + (0.067*P*)^2^ + 14.6225*P*] where *P* = (*F*_o_^2^ + 2*F*_c_^2^)/3
*S* = 1.07	(Δ/σ)_max_ = 0.001
1340 reflections	Δρ_max_ = 1.13 e Å^−^^3^
100 parameters	Δρ_min_ = −0.74 e Å^−^^3^
1 restraint	Absolute structure: Flack (1983), with how many Friedel pairs?
Primary atom site location: structure-invariant direct methods	Flack parameter: 0.42 (9)

### Special details

**Table:**

Geometry. All e.s.d.'s (except the e.s.d. in the dihedral angle between two l.s. planes) are estimated using the full covariance matrix. The cell e.s.d.'s are taken into account individually in the estimation of e.s.d.'s in distances, angles and torsion angles; correlations between e.s.d.'s in cell parameters are only used when they are defined by crystal symmetry. An approximate (isotropic) treatment of cell e.s.d.'s is used for estimating e.s.d.'s involving l.s. planes.
Refinement. Refinement of *F*^2^ against ALL reflections. The weighted *R*-factor *wR* and goodness of fit *S* are based on *F*^2^, conventional *R*-factors *R* are based on *F*, with *F* set to zero for negative *F*^2^. The threshold expression of *F*^2^ > σ(*F*^2^) is used only for calculating *R*-factors(gt) *etc*. and is not relevant to the choice of reflections for refinement. *R*-factors based on *F*^2^ are statistically about twice as large as those based on *F*, and *R*-factors based on ALL data will be even larger.

### Fractional atomic coordinates and isotropic or equivalent isotropic displacement parameters (Å^2^)

**Table:**

	*x*	*y*	*z*	*U*_iso_*/*U*_eq_	
Br1	1.2451 (2)	0.69710 (7)	0.3819 (16)	0.0745 (8)	
S1	0.6334 (4)	0.65747 (14)	0.8676 (16)	0.0407 (9)	
N2	0.4231 (13)	0.5675 (5)	0.884 (6)	0.051 (3)	
H2A	0.3952	0.5318	0.8379	0.061*	
H2B	0.3608	0.5908	0.9878	0.061*	
C7	0.9282 (17)	0.5595 (6)	0.404 (6)	0.044 (4)	
H7	0.9250	0.5204	0.3294	0.052*	
C6	1.0594 (18)	0.5939 (6)	0.352 (6)	0.043 (4)	
H6	1.1456	0.5786	0.2435	0.052*	
N1	0.6666 (18)	0.5508 (6)	0.627 (4)	0.047 (4)	
C1	0.5630 (19)	0.5869 (7)	0.795 (4)	0.046 (5)	
C2	0.802 (2)	0.5836 (7)	0.568 (4)	0.042 (4)	
C4	0.933 (2)	0.6779 (7)	0.634 (5)	0.046 (4)	
H4	0.9364	0.7170	0.7084	0.055*	
C3	0.804 (2)	0.6421 (8)	0.682 (4)	0.045 (4)	
C5	1.057 (2)	0.6520 (7)	0.469 (4)	0.052 (5)	

### Atomic displacement parameters (Å^2^)

**Table:**

	*U*^11^	*U*^22^	*U*^33^	*U*^12^	*U*^13^	*U*^23^
Br1	0.0513 (10)	0.0510 (9)	0.1212 (18)	−0.0117 (8)	0.005 (2)	0.005 (2)
S1	0.0394 (18)	0.0344 (16)	0.048 (2)	−0.0010 (15)	0.006 (3)	0.015 (3)
N2	0.041 (7)	0.041 (6)	0.071 (10)	−0.005 (5)	−0.012 (15)	−0.004 (11)
C7	0.045 (8)	0.033 (7)	0.053 (11)	0.000 (6)	0.007 (13)	−0.012 (11)
C6	0.052 (9)	0.038 (7)	0.040 (10)	−0.002 (6)	0.014 (14)	−0.007 (11)
N1	0.054 (9)	0.040 (7)	0.047 (9)	−0.001 (7)	0.010 (8)	−0.006 (7)
C1	0.043 (9)	0.037 (8)	0.059 (15)	−0.007 (7)	0.000 (9)	−0.009 (8)
C2	0.047 (10)	0.038 (9)	0.041 (10)	−0.005 (8)	0.011 (8)	−0.004 (8)
C4	0.052 (11)	0.035 (8)	0.051 (11)	0.002 (8)	0.005 (9)	−0.008 (8)
C3	0.050 (11)	0.044 (9)	0.040 (10)	−0.004 (8)	0.005 (8)	−0.001 (8)
C5	0.051 (10)	0.044 (9)	0.060 (16)	−0.001 (8)	0.004 (9)	0.002 (8)

### Geometric parameters (Å, º)

**Table:**

Br1—C5	1.944 (18)	C6—C5	1.39 (2)
S1—C3	1.686 (19)	C6—H6	0.9300
S1—C1	1.725 (16)	N1—C1	1.39 (2)
N2—C1	1.33 (2)	N1—C2	1.40 (2)
N2—H2A	0.8600	C2—C3	1.39 (2)
N2—H2B	0.8600	C4—C3	1.39 (3)
C7—C6	1.39 (2)	C4—C5	1.39 (2)
C7—C2	1.39 (2)	C4—H4	0.9300
C7—H7	0.9300		
			
C3—S1—C1	92.4 (9)	N1—C1—S1	113.3 (12)
C1—N2—H2A	120.0	C7—C2—C3	121.1 (16)
C1—N2—H2B	120.0	C7—C2—N1	122.0 (15)
H2A—N2—H2B	120.0	C3—C2—N1	116.9 (16)
C6—C7—C2	119.7 (14)	C3—C4—C5	116.3 (15)
C6—C7—H7	120.1	C3—C4—H4	121.8
C2—C7—H7	120.1	C5—C4—H4	121.8
C7—C6—C5	117.5 (15)	C4—C3—C2	120.7 (17)
C7—C6—H6	121.2	C4—C3—S1	130.0 (14)
C5—C6—H6	121.2	C2—C3—S1	109.2 (14)
C1—N1—C2	108.1 (14)	C6—C5—C4	124.6 (16)
N2—C1—N1	121.7 (14)	C6—C5—Br1	114.7 (13)
N2—C1—S1	125.0 (13)	C4—C5—Br1	120.7 (12)
			
C2—C7—C6—C5	0 (3)	C7—C2—C3—C4	0 (3)
C2—N1—C1—N2	179.8 (19)	N1—C2—C3—C4	−180.0 (17)
C2—N1—C1—S1	0.3 (18)	C7—C2—C3—S1	179.8 (17)
C3—S1—C1—N2	−179.9 (18)	N1—C2—C3—S1	0 (2)
C3—S1—C1—N1	−0.4 (15)	C1—S1—C3—C4	−180 (2)
C6—C7—C2—C3	0 (3)	C1—S1—C3—C2	0.4 (15)
C6—C7—C2—N1	−179.9 (19)	C7—C6—C5—C4	0 (3)
C1—N1—C2—C7	180 (2)	C7—C6—C5—Br1	−179.5 (15)
C1—N1—C2—C3	0 (2)	C3—C4—C5—C6	0 (3)
C5—C4—C3—C2	0 (3)	C3—C4—C5—Br1	179.6 (14)
C5—C4—C3—S1	−179.7 (15)		

### Hydrogen-bond geometry (Å, º)

**Table:**

*D*—H···*A*	*D*—H	H···*A*	*D*···*A*	*D*—H···*A*
N2—H2*B*···Br1^i^	0.86	3.04	3.864 (17)	160
N2—H2*A*···N1^ii^	0.86	2.26	2.94 (2)	136
C4—H4···Br1^iii^	0.93	2.87	3.402 (17)	118

Symmetry codes: (i) *x*−1, *y*, *z*+1; (ii) −*x*+1, −*y*+1, *z*+1/2; (iii) *x*−1/2, −*y*+3/2, *z*.
